# Author Correction: Adenosine triggers early astrocyte reactivity that provokes microglial responses and drives the pathogenesis of sepsis-associated encephalopathy in mice

**DOI:** 10.1038/s41467-024-52497-x

**Published:** 2024-09-18

**Authors:** Qilin Guo, Davide Gobbo, Na Zhao, Hong Zhang, Nana-Oye Awuku, Qing Liu, Li-Pao Fang, Tanja M. Gampfer, Markus R. Meyer, Renping Zhao, Xianshu Bai, Shan Bian, Anja Scheller, Frank Kirchhoff, Wenhui Huang

**Affiliations:** 1 Molecular Physiology, Center for Integrative Physiology and Molecular Medicine (CIPMM), University of Saarland, 66421 Homburg, Germany; 2 Center for Gender-specific Biology and Medicine (CGBM), University of Saarland, 66421 Homburg, Germany; 3 Institute of Anatomy and Cell Biology, University of Saarland, 66421 Homburg, Germany; 4 Biophysics, CIPMM, University of Saarland, 66421 Homburg, Germany; 5 Molecular Neurophysiology, CIPMM, University of Saarland, 66421 Homburg, Germany; 6 Department of Experimental and Clinical Toxicology, Institute of Experimental and Clinical Pharmacology and Toxicology, Center for Molecular Signaling (PZMS), University of Saarland, 66421 Homburg, Germany; 7 Institute for Regenerative Medicine, Shanghai East Hospital, Frontier Science Center for Stem Cell Research, School of Life Sciences and Technology, Tongji University, 200092 Shanghai, China

**Keywords:** Astrocyte, Neuroimmunology

Correction to: *Nature Communications* 10.1038/s41467-024-50466-y, published online 27 July 2024

The original version of this Article contained incorrect graphs in Figure 6h. In the original Figure 6h, the colour coding of the curves in the middle panel (6hpi) and right panel (24hpi) were inadvertently reversed. Specifically, the black curves in these panels should be red, representing Adora1 cKO, and the red curves should be black, representing the sham.

The correct version of Figure 6 is:
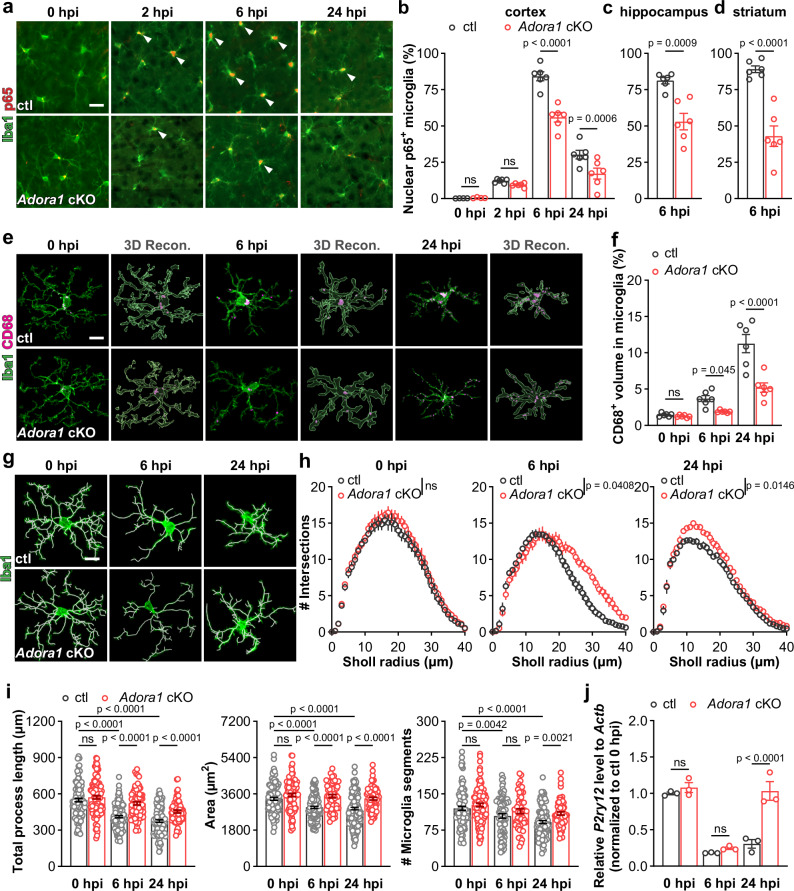


Which replaces the previous incorrect version:
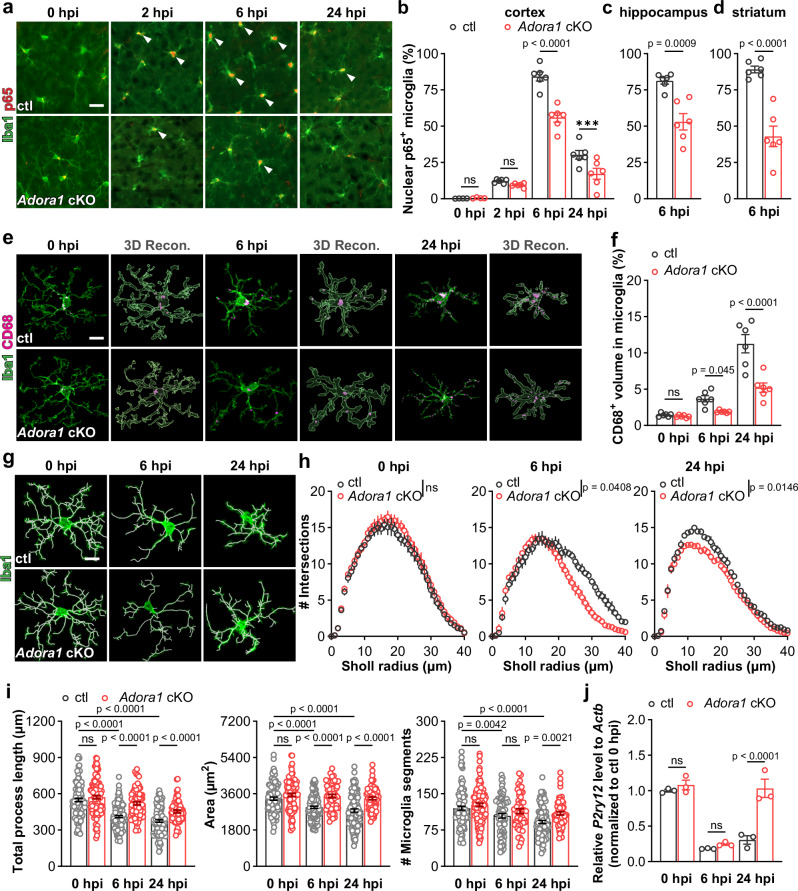


Figure 6 has been corrected in both the PDF and HTML versions of the Article.

