# Supplementary material for: Adenosine triggers early astrocyte reactivity that provokes microglial responses and drives the pathogenesis of sepsis-associated encephalopathy in mice
Source: Nat Commun. 2024 Jul 27;15:6340. doi: 10.1038/s41467-024-50466-y (PMC11283516; doi:10.1038/s41467-024-50466-y)
Supplement: Supplementary file 1 — Supplementary Information [file 41467_2024_50466_MOESM1_ESM.pdf]

Adenosine triggers early astrocyte reactivity that provokes microglial responses and drives the pathogenesis of sepsis-associated encephalopathy in mice

Qilin Guo[1,2], Davide Gobbo[1], Na Zhao[1,3], Hong Zhang[4], Nana-Oye Awuku[5], Qing Liu[1], Li-Pao Fang[1,2], Tanja M. Gampfer[6], Markus R. Meyer[6], Renping Zhao[4], Xianshu Bai[1,2], Shan Bian[7], Anja Scheller[1,2], Frank Kirchhoff[1,2]*, Wenhui Huang[1,2]*

1. Molecular Physiology, Center for Integrative Physiology and Molecular Medicine (CIPMM), University of Saarland, 66421, Homburg, Germany
2. Center for Gender-specific Biology and Medicine (CGBM), University of Saarland, 66421 Homburg, Germany
3. Institute of Anatomy and Cell Biology, University of Saarland, 66421, Homburg, Germany
4. Biophysics, CIPMM, University of Saarland, 66421 Homburg, Germany
5. Molecular Neurophysiology, CIPMM, University of Saarland, 66421 Homburg, Germany
6. Department of Experimental and Clinical Toxicology, Institute of Experimental and Clinical Pharmacology and Toxicology, Center for Molecular Signaling (PZMS), University of Saarland, 66421, Homburg, Germany
7. Institute for Regenerative Medicine, Shanghai East Hospital, Frontier Science Center for Stem Cell Research, School of Life Sciences and Technology, Tongji University, 200092, Shanghai, China

(*) For correspondence:

Frank Kirchhoff: Molecular Physiology, Center for Integrative Physiology and Molecular Medicine (CIPMM), University of Saarland, 66421 Homburg, Germany

Email: frank.kirchhoff@uks.eu

Wenhui Huang: Molecular Physiology, Center for Integrative Physiology and Molecular Medicine (CIPMM), University of Saarland, 66421 Homburg, Germany

Email: wenhui.huang@uks.eu

Supplementary information in this PDF file contains Supplementary Figures 1–11 with legends and Supplementary Table 1.

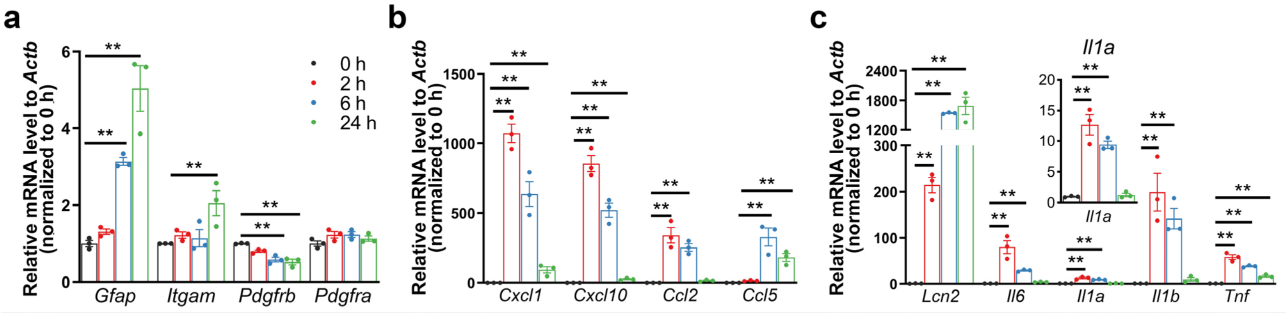

Supplementary Fig. 1: Peripheral LPS challenge evokes rapid neuroinflammatory responses and glial reaction.

a Changes of marker genes for astrocytes (*Gfap*), microglia (*Itgam*), pericytes (*Pdgfrb*), and OPCs (*Pdgfra*) in the cortex after PBS/LPS injection (n = 3 mice per group).

b, c Expression levels of several chemokines and proinflammatory cytokines in the cortex after PBS/LPS injection (n = 3 mice per group).

Summary data are presented as the mean ± SEM. Statistical significance in a-c were assessed by two-way ANOVA, Fisher's LSD test; $*P < 0.05$, $**P < 0.01$, $***P < 0.001$. Source data are provided as a Source Data file.

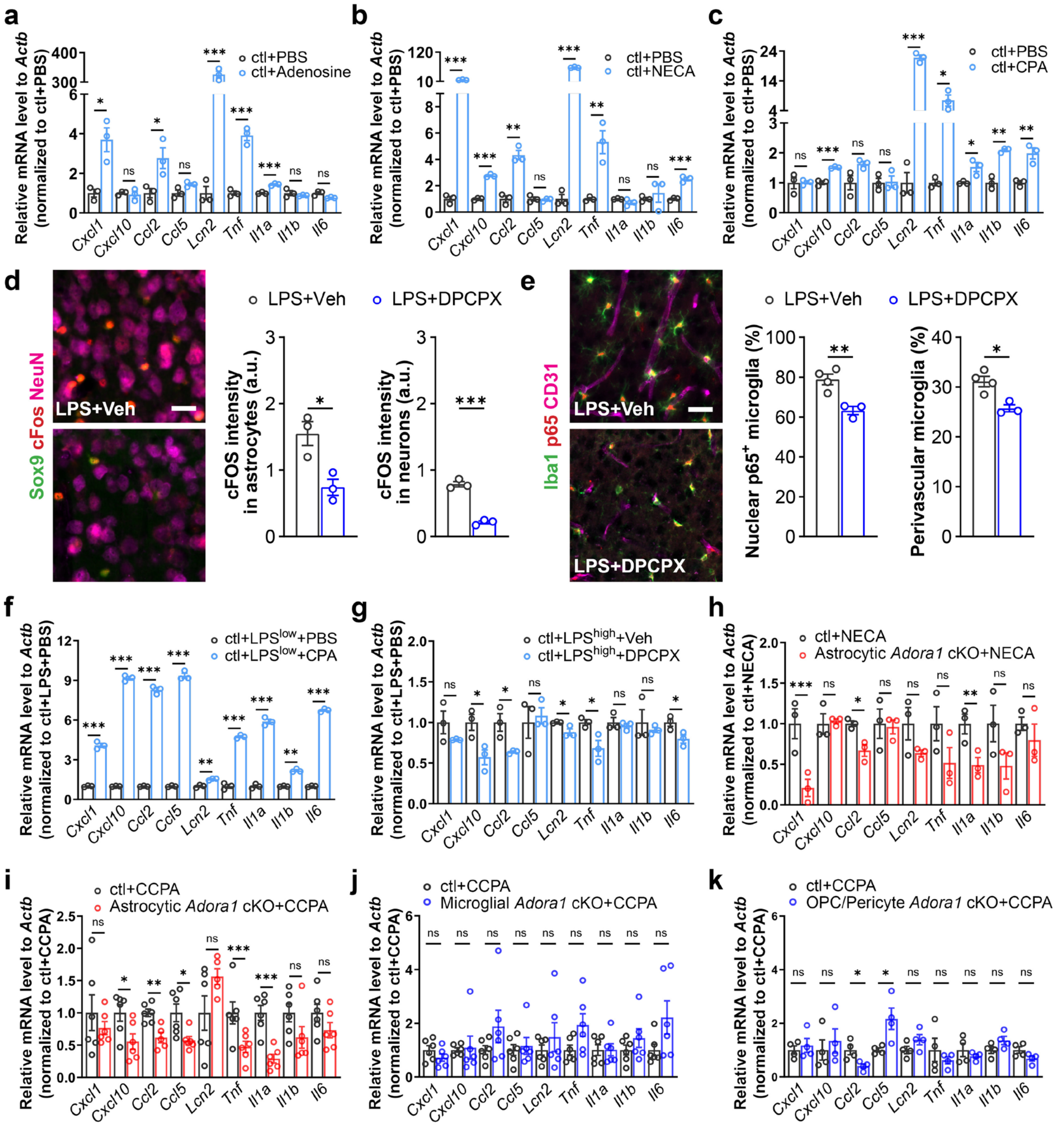

Supplementary Fig. 2: Adenosine evokes upregulation of inflammation-related genes in the brain via A1AR signaling.

a-c Expression of inflammation-related genes were enhanced in the mouse cortex six hours post adenosine, NECA, and CPA injections (n = 3 mice per group).

d Representative images of immunoreactivity of c-Fos in Sox9^{+} astrocytes and NeuN+ neurons in the mouse cortex upon LPS and A1AR antagonist (DPCPX) injection (left). c-Fos expression in astrocytes and neurons was reduced by DPCPX (right) (n = 3 mice per group).

e Representative images of immunolabeled nuclear p65^{+} microglia and CD31^{+} blood vessels upon LPS and A1AR antagonist (DPCPX) injection (left). Nuclear p65^{+} microglia and perivascular microglia were reduced by DPCPX (right) (n = 4 mice in LPS+Veh group, n = 3 mice in LPS+DPCPX group).

f CPA further upregulated the inflammation-related genes in the cortex induced by a peripheral LPSlow (1 mg/kg, i.p.) injection. (n = 3 mice per group).

g DPCPX administration reduced the inflammation-related genes in the cortex induced by a peripheral LPShigh (5mg/kg, i.p.) injection. (n = 3 mice per group).

h, i Inflammation-related gene expressions were reduced in the cortex of *Adora1* cKO mice at 6 hours post NECA and CCPA injection compared to ctl mice (n = 3 mice per group in (G), n = 6 mice per group in (H)).

j, k Inflammation-related gene expressions were not altered in the cortex of mice with specific ablation of *Adora1* in microglia (using Cx3CR1-CreERT2 mice) and oligodendrocyte precursor cells/pericytes (using NG2-CreERT2 mice, only Ccl2 was reduced).

Summary data are presented as the mean ± SEM. Statistical significance in **a-k** was assessed by two-tailed unpaired Student's t test; *P < 0.05, **P < 0.01, ***P < 0.001. Source data are provided as a Source Data file.

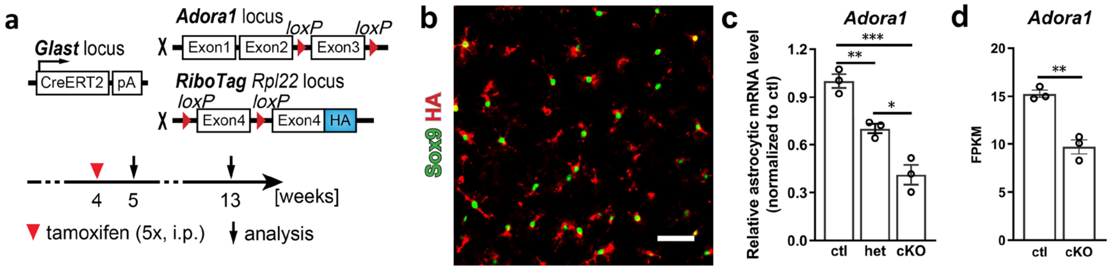

Supplementary Fig. 3: Generation and validation of astrocyte-specific A1AR deficient mice.

a Schematic illustration of mouse breeding for astrocyte-specific A1AR deficient mice (*Adora1* cKO) and experiment plan. GLAST-CreERT2 (GLAC) mice were crossed to floxed *Adora1* mice. RiboTag mice were also introduced to the breeding for specifically and directly purify translated mRNA from astrocytes without sorting cells.

b Representative image of RiboTag expression (indicated by HA-tag) in Sox9^{+} astrocytes. Scale bar = 50 µm.

c *Adora1* expression in astrocytes was reduced in *Adora1*$^{fl/wt}$ (het) and *Adora1* cKO mice one week after tamoxifen injection by using qPCR (n = 3 mice per group).

d *Adora1* expression in astrocytes was reduced in *Adora1* cKO mice 9 weeks after tamoxifen injection by using RNA-Seq (n = 3 mice per group).

Summary data are presented as the mean ± SEM. Statistical significance in **c** was assessed using a one-way ANOVA, Fisher's LSD test; statistical significance in d was assessed using two tailed unpaired Student’s t test, *P < 0.05, **P < 0.01, ***P < 0.001. Source data are provided as a Source Data file.

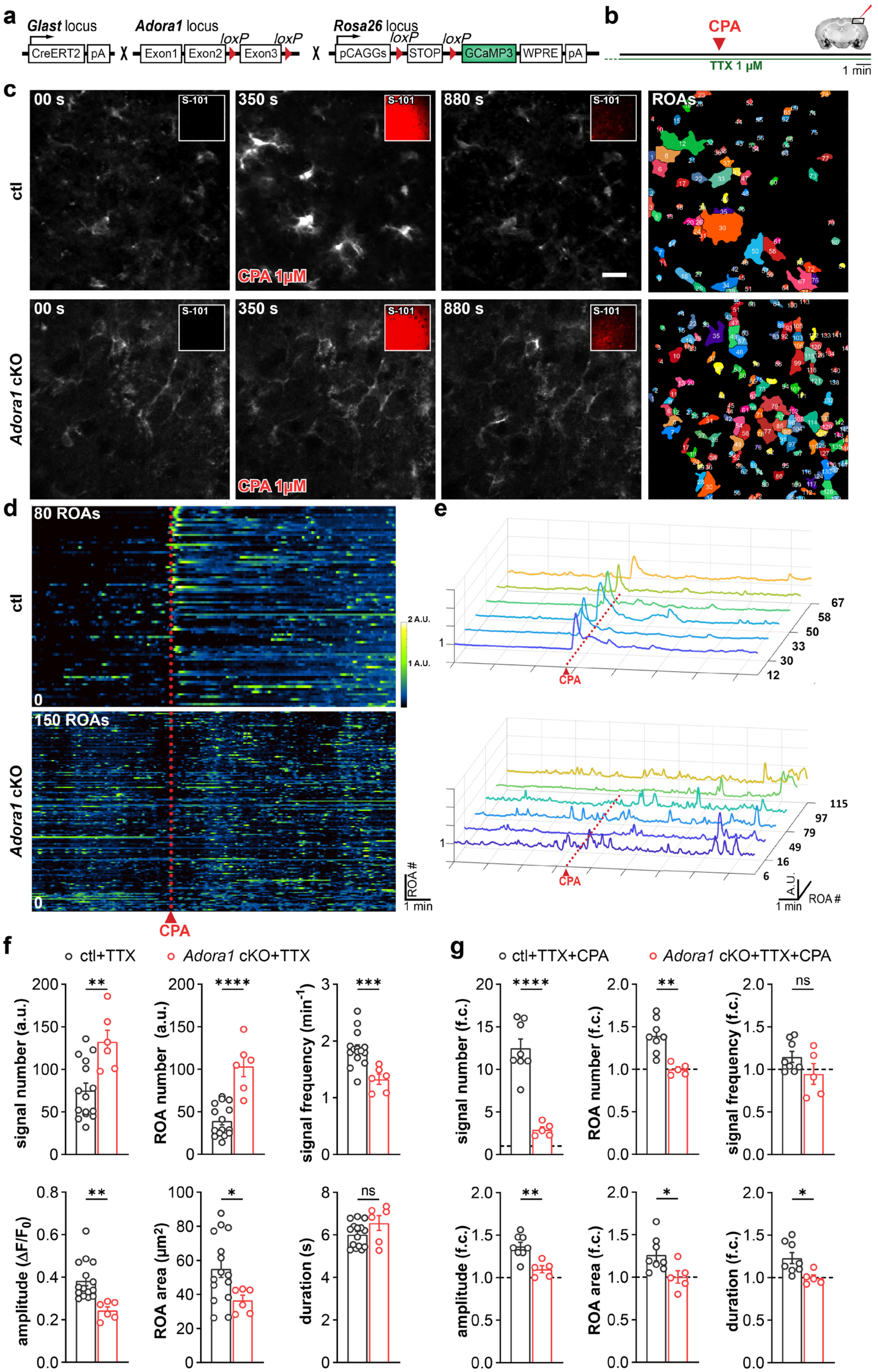
a
Glast locus
Adora1 locus
Rosa26 locus
CreERT2
pA
Exon1
Exon2
Exon3
loxP
pCAGGs
STOP
GCaMP3
WPRE
b
CPA
TTX 1 μM
1 min
c
ctl
Adora1 cKO
00 s
350 s
880 s
S-101
CPA 1μM
ROAs
d
80 ROAs
150 ROAs
2 A.U.
1 A.U.
ROA #
1 min
CPA
e
f
ctl+TTX
Adora1 cKO+TTX
signal number (a.u.)
ROA number (a.u.)
signal frequency (min⁻¹)
amplitude (ΔF/F0)
ROA area (μm2)
duration (s)
g
ctl+TTX+CPA
Adora1 cKO+TTX+CPA
signal number (f.c.)
ROA number (f.c.)
signal frequency (f.c.)
amplitude (f.c.)
ROA area (f.c.)
duration (f.c.)
ns

Supplementary Fig. 4: Functional validation of astrocyte-specific A1AR deficient mice by Ca^{2+} imaging.

a, b Schematic illustration of mouse breeding for Ca^{2+} imaging and experiment plan. GLACxA1AR$^{fl/fl}$ mice were crossed to Rosa26-GCaMP3 mice. GLACxA1AR$^{fl/fl}$xRosa26-GCaMP3 mice were treated with tamoxifen at 4 weeks and used for *ex vivo* Ca^{2+} imaging at 13 weeks of age. Coronal brain slices were incubated in TTX (tetrodotoxin). During recording the A1AR agonist CPA (1 µM) was applied focally. Sulforhodamine 101 (SR101, 4 µg/ml) was mixed with CPA to indicate the drug application.

c Images showing the change of Ca^{2+} activity during the recording in ctl and *Adora1 cKO*mice. Notably, CPA application evoked high Ca^{2+} increase in ctl mice which was not observed in *Adora1* cKO mice, functionally confirming the deletion of A1ARs in astrocytes. The rightmost images show automatically detected regions of interests (ROIs) with dynamic Ca^{2+} activities by a custom-made tool MSparkles.

d Heatmap plot showing amplitude and duration of spontaneous Ca^{2+} events detected from all ROIs.

e Six ROIs were selected to show the characteristics of Ca^{2+} events.

f, g Analyses of Ca^{2+} events in ctl (n = 15 slices from 3 mice) and *Adora1* cKO (n = 6 slices from 3 mice) slices mice before (as baseline) and after CPA application using MSparkles.

Summary data are presented as the mean ± SEM. Statistical significance in **f, g** was assessed using two tailed unpaired Student's t test, *P < 0.05, **P < 0.01, ***P < 0.001, ****P < 0.0001. Source data are provided as a Source Data file.

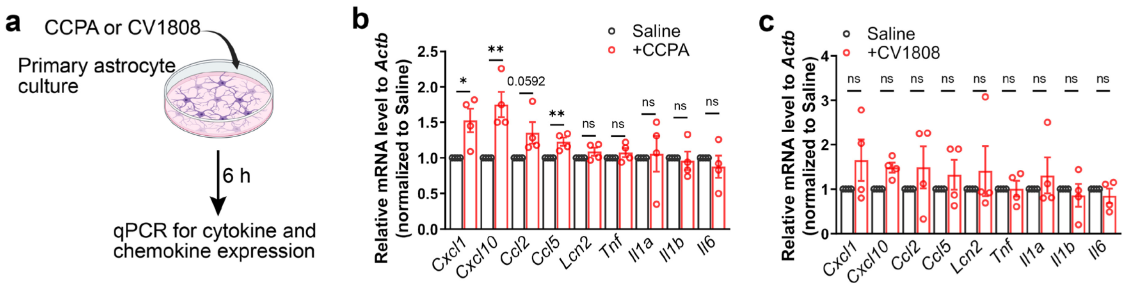

Supplementary Fig. 5: Activation of A1AR evokes upregulation of inflammation-related genes in primary astrocytes.

a Schematic illustration of drug application to primary astrocytes. Created with BioRender.com released under a Creative Commons Attribution-NonCommercial-NoDerivs 4.0 International license.

b, c CCPA application significantly increased expression levels of several chemokines (e.g., *Cxcl1*, *Cxcl10*, *Ccl5*), whereas CV1808 (non-selective antagonist of A2ARs) did not cause significant expression alterations of all the tested inflammation-related genes.

Summary data are presented as the mean ± SEM, n = 4 individual primary cultures. Statistical significance of each gene expression in **b, c** was assessed using two tailed unpaired Student's t test, ns: not significant, *P < 0.05, **P < 0.01. Source data are provided as a Source Data file.

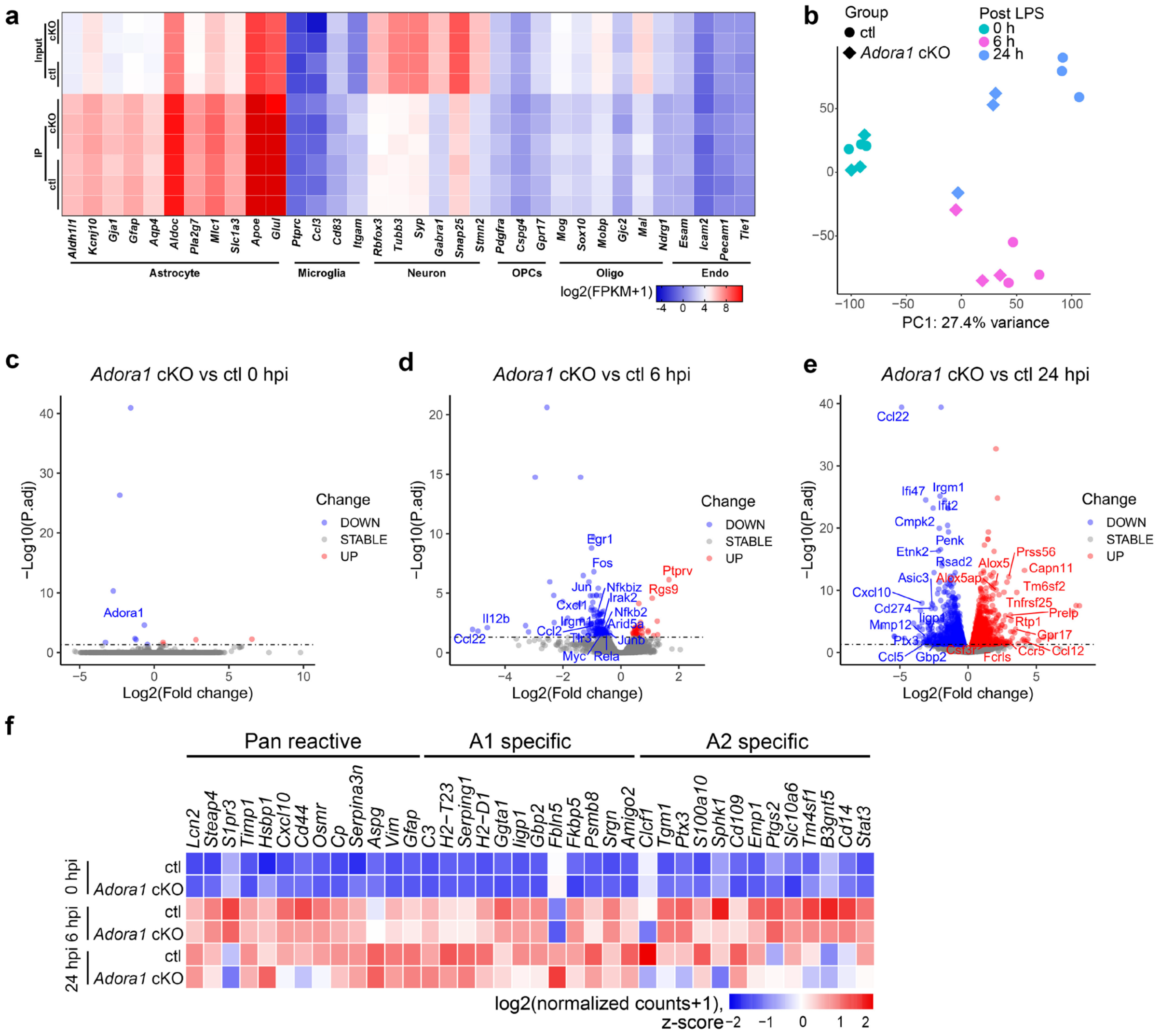

Supplementary Fig. 6: A1AR-deficient astrocytes are less reactive to the peripheral LPS challenge.

a Heatmap of cell type-specific marker gene expression showed immunoprecipitation (IP) of RiboTag enriched astrocyte-specific genes.
b PCA (principal component analysis) of the RNA-seq dataset (n = 3 mice per group).
c-e Volcano plots showing gene expression changes in *Adora1* cKO group compared to ctl group at 0 hpi, 6 hpi, 24 hpi.
f Heatmap of marker genes for A1/A2 astrocytes.

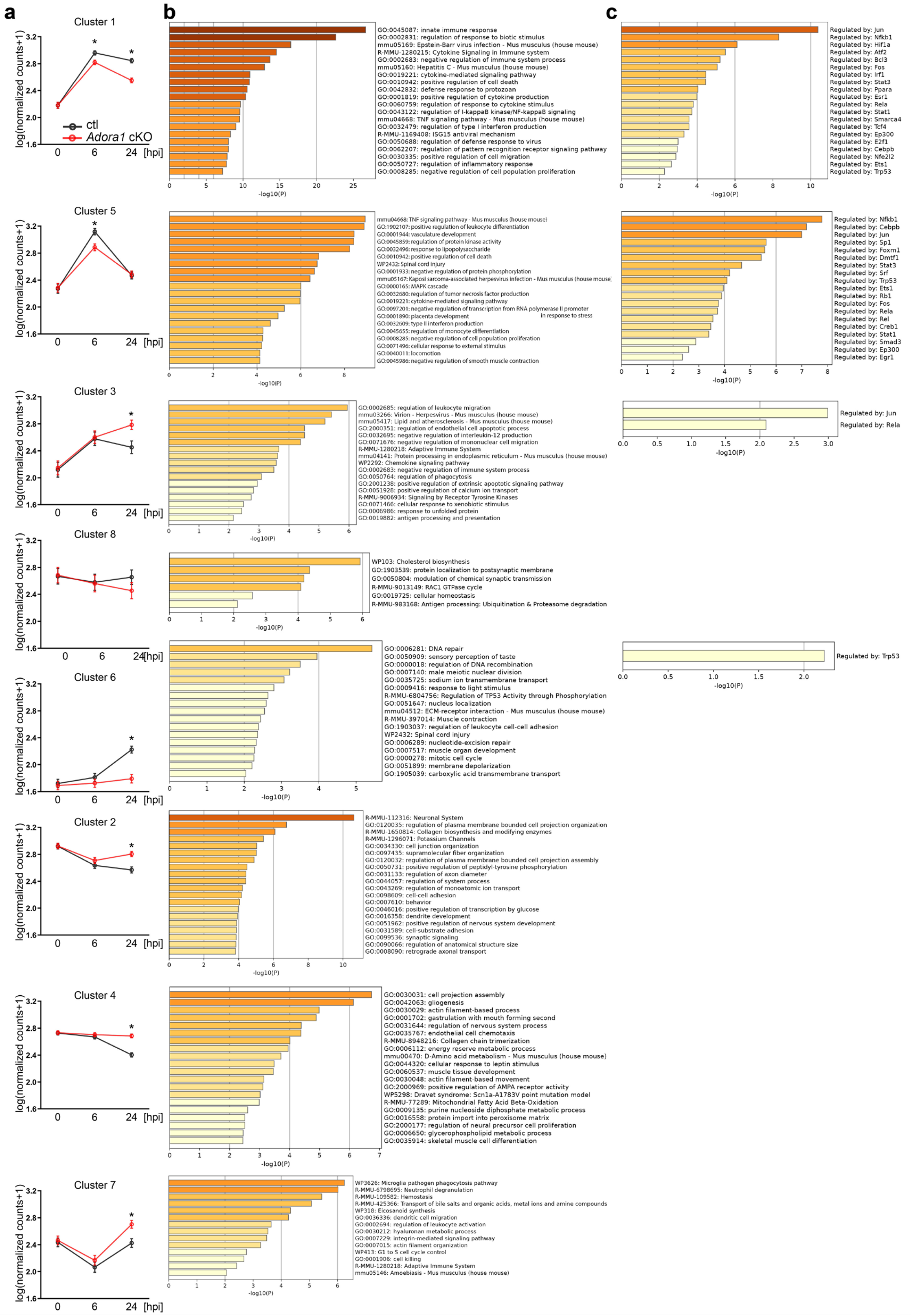

a
b
c
Cluster 1
log(normalized counts+1)
ctl
Adora1 cKO
0 6 24 [hpi]
GO:0045087: innate immune response
GO:0002831: regulation of response to biotic stimulus
mmu05169: Epstein-Barr virus infection - Mus musculus (house mouse)
R-MMU-1280215: Cytokine Signaling in Immune system
GO:0002683: negative regulation of immune system process
mmu05160: Hepatitis C - Mus musculus (house mouse)
GO:0019221: cytokine-mediated signaling pathway
GO:0010942: positive regulation of cell death
GO:0042832: defense response to protozoan
GO:0001819: positive regulation of cytokine production
GO:0060759: regulation of response to cytokine stimulus
GO:0043122: regulation of I-kappaB kinase/NF-kappaB signaling
mmu04668: TNF signaling pathway - Mus musculus (house mouse)
GO:0032479: regulation of type I interferon production
R-MMU-1169408: ISG15 antiviral mechanism
GO:0050688: regulation of defense response to virus
GO:0062207: regulation of pattern recognition receptor signaling pathway
GO:0030335: positive regulation of cell migration
GO:0050727: regulation of inflammatory response
GO:0008285: negative regulation of cell population proliferation
-log10(P)
Regulated by: Jun
Regulated by: Nfkb1
Regulated by: Hif1a
Regulated by: Atf2
Regulated by: Bcl3
Regulated by: Fos
Regulated by: Irf1
Regulated by: Stat3
Regulated by: Ppara
Regulated by: Esr1
Regulated by: Rela
Regulated by: Stat1
Regulated by: Smarca4
Regulated by: Tcf4
Regulated by: Ep300
Regulated by: E2f1
Regulated by: Cebpb
Regulated by: Nfe2l2
Regulated by: Ets1
Regulated by: Trp53
Cluster 5
mmu04668: TNF signaling pathway - Mus musculus (house mouse)
GO:1902107: positive regulation of leukocyte differentiation
GO:0001944: vasculature development
GO:0045859: regulation of protein kinase activity
GO:0032496: response to lipopolysaccharide
GO:0010942: positive regulation of cell death
WP2432: Spinal cord injury
GO:0001933: negative regulation of protein phosphorylation
mmu05167: Kaposi sarcoma-associated herpesvirus infection - Mus musculus (house mouse)
GO:0000165: MAPK cascade
GO:0032680: regulation of tumor necrosis factor production
GO:0019221: cytokine-mediated signaling pathway
GO:0097201: negative regulation of transcription from RNA polymerase II promoter in response to stress
GO:0001890: placenta development
GO:0032609: type II interferon production
GO:0045655: regulation of monocyte differentiation
GO:0008285: negative regulation of cell population proliferation
GO:0071496: cellular response to external stimulus
GO:0040011: locomotion
GO:0045986: negative regulation of smooth muscle contraction
Regulated by: Nfkb1
Regulated by: Cebpb
Regulated by: Jun
Regulated by: Sp1
Regulated by: Foxm1
Regulated by: Dmtf1
Regulated by: Stat3
Regulated by: Srf
Regulated by: Trp53
Regulated by: Ets1
Regulated by: Rb1
Regulated by: Fos
Regulated by: Rela
Regulated by: Rel
Regulated by: Creb1
Regulated by: Stat1
Regulated by: Smad3
Regulated by: Ep300
Regulated by: Egr1
Cluster 3
GO:0002685: regulation of leukocyte migration
mmu03266: Virion - Herpesvirus - Mus musculus (house mouse)
mmu05417: Lipid and atherosclerosis - Mus musculus (house mouse)
GO:2000351: regulation of endothelial cell apoptotic process
GO:0032695: negative regulation of interleukin-12 production
GO:0071676: negative regulation of mononuclear cell migration
R-MMU-1280218: Adaptive Immune System
mmu04141: Protein processing in endoplasmic reticulum - Mus musculus (house mouse)
WP2292: Chemokine signaling pathway
GO:0002683: negative regulation of immune system process
GO:0050764: regulation of phagocytosis
GO:2001238: positive regulation of extrinsic apoptotic signaling pathway
GO:0051928: positive regulation of calcium ion transport
R-MMU-9006934: Signaling by Receptor Tyrosine Kinases
GO:0071466: cellular response to xenobiotic stimulus
GO:0006986: response to unfolded protein
GO:0019882: antigen processing and presentation
Regulated by: Jun
Regulated by: Rela
Cluster 8
WP103: Cholesterol biosynthesis
GO:1903539: protein localization to postsynaptic membrane
GO:0050804: modulation of chemical synaptic transmission
R-MMU-9013149: RAC1 GTPase cycle
GO:0019725: cellular homeostasis
R-MMU-983168: Antigen processing: Ubiquitination & Proteasome degradation
Cluster 6
GO:0006281: DNA repair
GO:0050909: sensory perception of taste
GO:0000018: regulation of DNA recombination
GO:0007140: male meiotic nuclear division
GO:0035725: sodium ion transmembrane transport
GO:0009416: response to light stimulus
R-MMU-6804756: Regulation of TP53 Activity through Phosphorylation
GO:0051647: nucleus localization
mmu04512: ECM-receptor interaction - Mus musculus (house mouse)
R-MMU-397014: Muscle contraction
GO:1903037: regulation of leukocyte cell-cell adhesion
WP2432: Spinal cord injury
GO:0006289: nucleotide-excision repair
GO:0007517: muscle organ development
GO:0000278: mitotic cell cycle
GO:0051899: membrane depolarization
GO:1905039: carboxylic acid transmembrane transport
Regulated by: Trp53
Cluster 2
R-MMU-112316: Neuronal System
GO:0120035: regulation of plasma membrane bounded cell projection organization
R-MMU-1650814: Collagen biosynthesis and modifying enzymes
R-MMU-1296071: Potassium Channels
GO:0034330: cell junction organization
GO:0097435: supramolecular fiber organization
GO:0120032: regulation of plasma membrane bounded cell projection assembly
GO:0050731: positive regulation of peptidyl-tyrosine phosphorylation
GO:0031133: regulation of axon diameter
GO:0044057: regulation of system process
GO:0043269: regulation of monoatomic ion transport
GO:0098609: cell-cell adhesion
GO:0007610: behavior
GO:0046016: positive regulation of transcription by glucose
GO:0016358: dendrite development
GO:0051962: positive regulation of nervous system development
GO:0031589: cell-substrate adhesion
GO:0099536: synaptic signaling
GO:0090066: regulation of anatomical structure size
GO:0008090: retrograde axonal transport
Cluster 4
GO:0030031: cell projection assembly
GO:0042063: gliogenesis
GO:0030029: actin filament-based process
GO:0001702: gastrulation with mouth forming second
GO:0031644: regulation of nervous system process
GO:0035767: endothelial cell chemotaxis
R-MMU-8948216: Collagen chain trimerization
GO:0006112: energy reserve metabolic process
mmu00470: D-Amino acid metabolism - Mus musculus (house mouse)
GO:0044320: cellular response to leptin stimulus
GO:0060537: muscle tissue development
GO:0030048: actin filament-based movement
GO:2000969: positive regulation of AMPA receptor activity
WP5298: Dravet syndrome: Scn1a-A1783V point mutation model
R-MMU-77289: Mitochondrial Fatty Acid Beta-Oxidation
GO:0009135: purine nucleoside diphosphate metabolic process
GO:0016558: protein import into peroxisome matrix
GO:2000177: regulation of neural precursor cell proliferation
GO:0006650: glycerophospholipid metabolic process
GO:0035914: skeletal muscle cell differentiation
Cluster 7
WP3626: Microglia pathogen phagocytosis pathway
R-MMU-6798695: Neutrophil degranulation
R-MMU-109582: Hemostasis
R-MMU-425366: Transport of bile salts and organic acids, metal ions and amine compounds
WP318: Eicosanoid synthesis
GO:0036336: dendritic cell migration
GO:0002694: regulation of leukocyte activation
GO:0030212: hyaluronan metabolic process
GO:0007229: integrin-mediated signaling pathway
GO:0007015: actin filament organization
WP413: G1 to S cell cycle control
GO:0001906: cell killing
R-MMU-1280218: Adaptive Immune System
mmu05146: Amoebiasis - Mus musculus (house mouse)

Supplementary Fig. 7: Transcriptomic data analysis reveals A1AR-deficient astrocytes are less reactive to the peripheral LPS challenge.

a Mean profile representation of the temporal gene expression pattern for each cluster in **Fig. 4a**. Data points correspond to 0 hpi, 6 hpi, 24 hpi.
b Metascape pathways for each cluster in **Fig. 4a** generated by Metascape analysis.
c List of prediction of transcription regulators following expression pattern of sub-clusters in **Fig. 4a**.

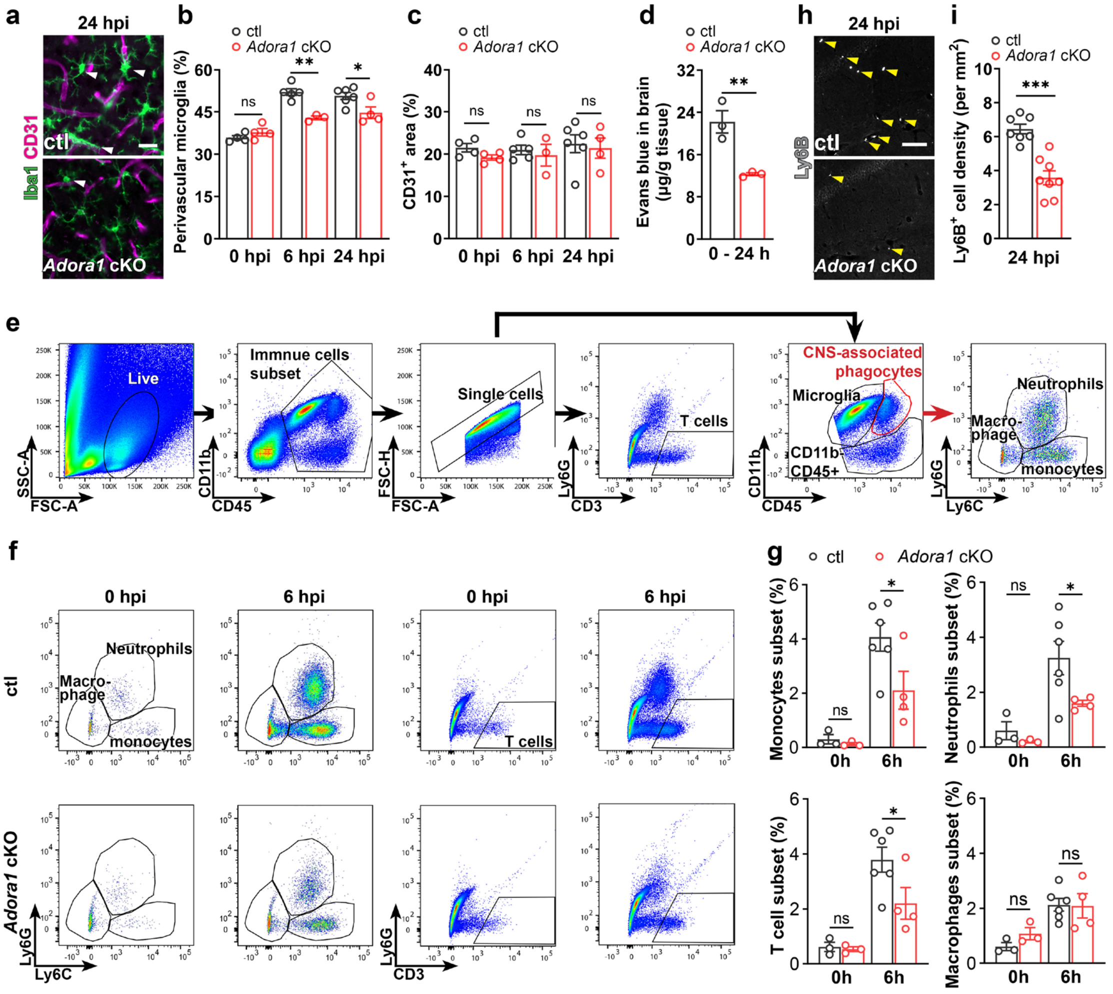

Supplementary Fig. 8: Astrocytic A1AR deficiency reduces BBB disruption and neutrophil infiltration post peripheral LPS injection.

a Representative images of immunolabeled Iba1^{+} microglia and CD31^{+} blood vessels at 24 hpi. Arrowheads indicate perivascular microglia. Scale bar = 20 µm.
b Proportion of perivascular microglia was increased in *Adora1* cKO mice compared to ctl mice (n = 4 mice in ctl and *Adora1* cKO at 0 hpi, n = 5 mice in ctl at 6 hpi, n = 3 mice in *Adora1* cKO at 6 hpi, n = 6 mice in ctl at 24 hpi, n = 4 mice in *Adora1* cKO at 24 hpi).
c CD31^{+} area was not altered in *Adora1* cKO and ctl mice at 0 hpi, 6 hpi, 24 hpi.
d EB extravasation was reduced in the brains of *Adora1* cKO mice compared to ctl mice which were injected with EB at 0 hpi and analyzed at 24 hpi (n = 3 mice per group).
e Gating strategy for different cell types from brain cell suspension.
f Proportion of infiltrated immune cell (monocytes, neutrophils, and T cells) was reduced in the brain of cKO mice at 6 hpi, except macrophages.
g Statistical analysis of the ratio of specific immune cell subtypes in the immune cell subset (single cells). One dot stands for one mouse. (n = 3 ctl and *Adora1* cKO mice at 0 hpi; n = 6 ctl mice and 4 *Adora1* cKO mice at 6 hpi).
h Representative images of immunolabeled of Ly6B^{+} neutrophils in the brain parenchyma at 24 hpi. Scale bars = 20 µm.
i The density of Ly6B^{+} cells was reduced in the brain of *Adora1* cKO mice compared to ctl mice at 24 hpi (n = 7 ctl mice and 8 *Adora1* cKO mice).
Summary data are presented as the mean ± SEM. Statistical significance in **b, c, g** were assessed using a two-way ANOVA, Fisher's LSD test. Statistical significance in **d, i** was assessed using

two-tailed unpaired Student’s t test, ns: not significant, *P < 0.05, **P < 0.01, ***P < 0.001. Source data are provided as a Source Data file.

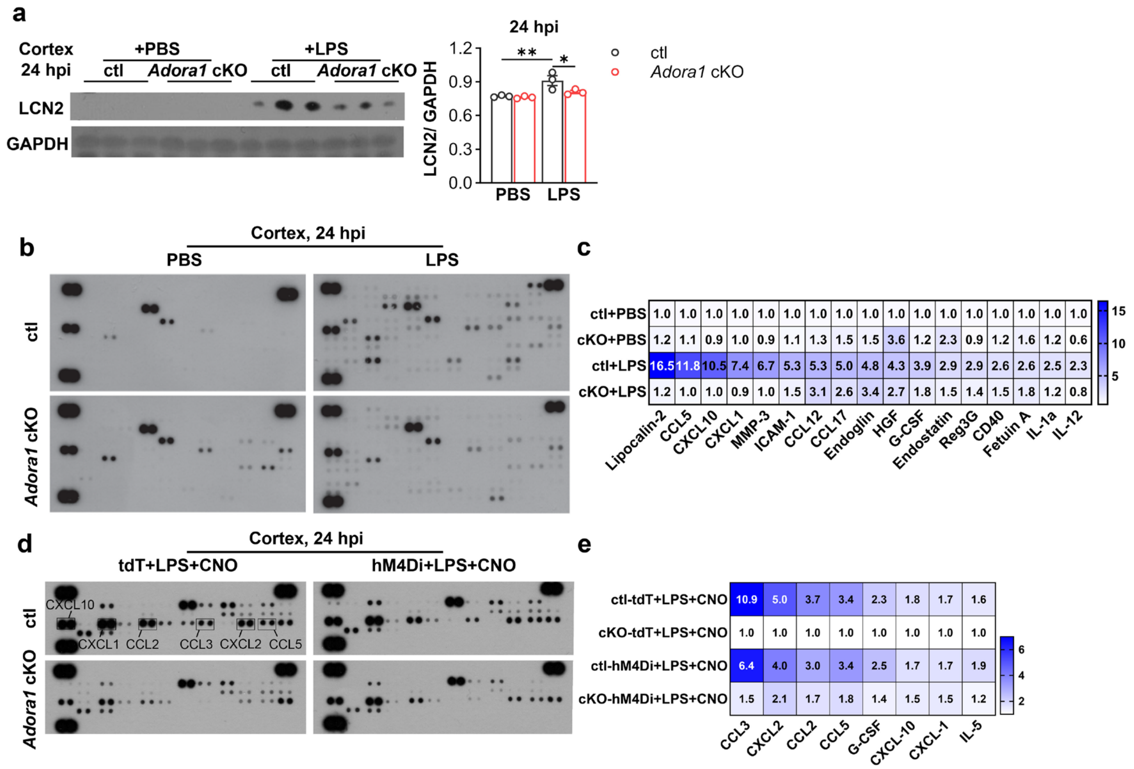

Supplementary Fig. 9: Astrocytic A1AR deficiency reduces overall neuroinflammation upon LPS challenge which is attenuated by enhancing Gi signaling.

a LCN2 expression was reduced in *Adora1* cKO compared to ctl 24 hpi by Western blot.
b, c The expression of 111 cytokines in the cortex of ctl and *Adora1* cKO mice was measured by a proteomic profiling assay at 24 h after PBS or LPS injection. Cytokine expression was reduced in the cortex of *Adora1* cKO group compared to ctl (PBS) group (samples from 3 mice were mixed for each group).
d, e The expression of 40 cytokines in the cortex of AAV-infected ctl and *Adora1* cKO mice was measured by a proteomic profiling assay 24 hours after LPS and CNO injection. Enhancing Gi signaling in *Adora1* cKO mice increased cytokine expression after LPS and CNO injection (samples from 3 mice were pooled for each group).
Summary data are presented as the mean ± SEM. Statistical significance in **a** were assessed by two-way ANOVA, Fisher's LSD test, ns: not significant, $*P < 0.05$, $**P < 0.01$. Source data are provided as a Source Data file.

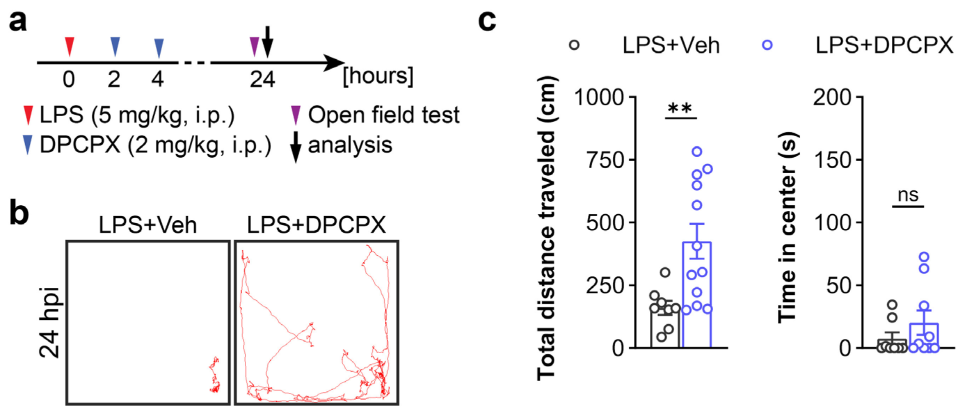

Supplementary Fig. 10: A1AR antagonist treatment ameliorated depression-like behavior of mice post LPS injection.

a Schematic illustration. Ctl mice were injected with DPCPX at the early phase of systemic inflammation and were used in open-field test at 24 h post LPS injection.

b Representative trajectory analysis of Vehicle (Veh) and DPCPX-treated mice in 10 min in the open-field test at 24 hpi.

c DPCPX treated mice displayed increased locomotion compared to vehicle ctl mice at 24 hpi (n = 8 mice in LPS+Veh group, n = 12 mice in LPS+DPCPX group).

Summary data are presented as the mean ± SEM in **c**. Statistical significance in **c** was assessed using two-tailed unpaired Student's t test. ns: not significant, **P < 0.01. Source data are provided as a Source Data file.

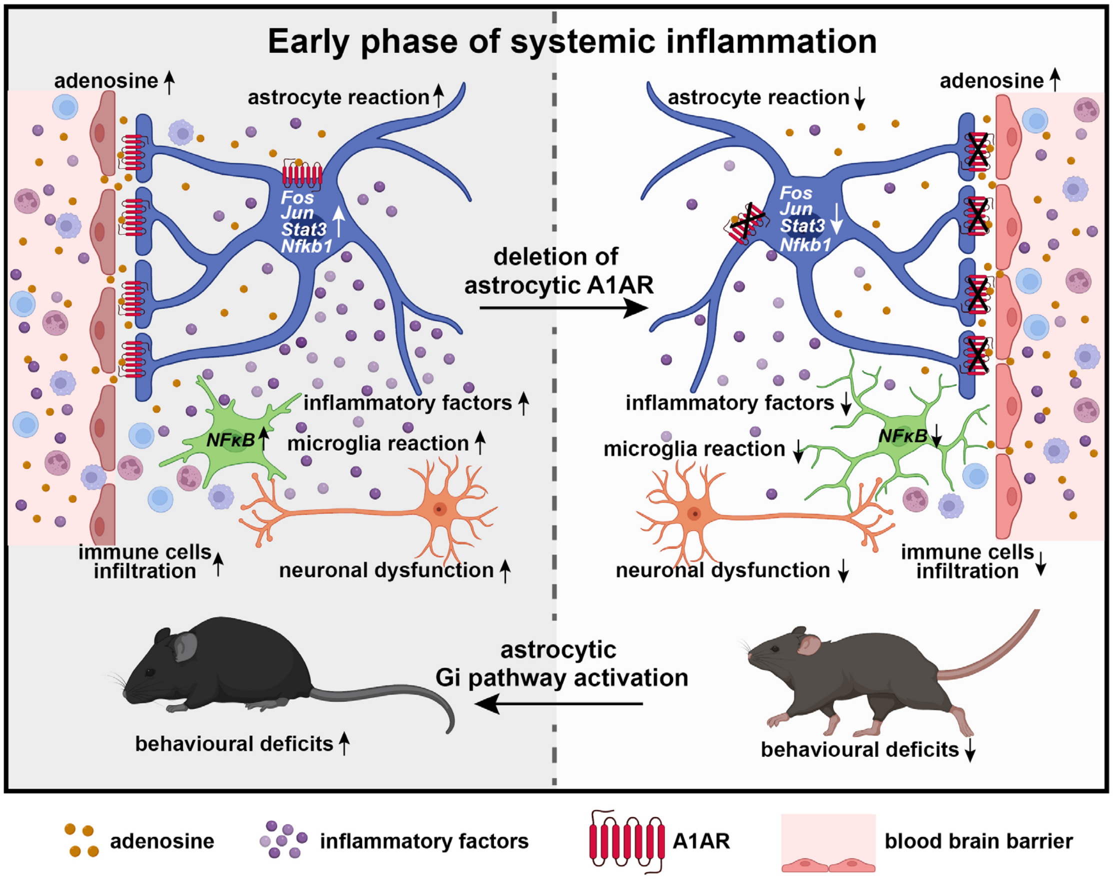

Supplementary Fig. 11: Graphical abstract.

Graphical abstract was created with BioRender.com released under a Creative Commons Attribution-NonCommercial-NoDerivs 4.0 International license.

Supplementary Table 1: key resources

REAGENT or RESOURCE	SOURCE	IDENTIFIER
**Antibodies**		
Goat anti-Sox9 (1:500)	R&D Systems	Cat# AF3075
Rabbit anti-Iba1 (1:1000)	Wako	Cat# 019-19741
Goat anti-Iba1 (1:500)	abcam	Cat# ab5076
Mouse anti-NeuN (1:500)	Millipore	Cat# MAB377
Rat anti-CD31 (1:100)	BD Pharmingen	Cat# 550274
Rat anti-Ly6B (1:500)	Bio Rad	Cat# MCA771GT
Mouse anti-HA (1:500)	Biolegend	Cat# 901513
Goat anti-LCN2 (1:1000)	R&D Systems	Cat# AF1857
Chicken anti-GFP (1:1000)	Thermo Fisher Scientific	Cat# 10524234
Rabbit anti-p-STAT3 (1:1000)	Cell Signaling Technology	Cat# 9145
Rabbit anti-NF-kappaB p65 (1:500)	Cell Signaling Technology	Cat# 8242
Guinea pig anti-cFos (1:4000)	Synaptic Systems	Cat# 226004
Brilliant Violet 421™ anti-mouse CD45 Antibody	Biolegend	Cat# 103133
APC anti-mouse Ly-6G Antibody	Biolegend	Cat# 127613
PerCP anti-mouse/human CD11b Antibody	Biolegend	Cat# 101229
PE/Cyanine7 anti-mouse Ly-6C Antibody	Biolegend	Cat# 128017
APC/Cyanine7 anti-mouse CD3 Antibody	Biolegend	Cat# 100221
Brilliant Violet 421™ Rat IgG2b, κ Isotype Ctrl Antibody	Biolegend	Cat# 400639
APC Rat IgG2a, κ Isotype Ctrl Antibody	Biolegend	Cat# 400511
PerCP Rat IgG2b, κ Isotype Ctrl Antibody	Biolegend	Cat# 400629
PE/Cyanine7 Rat IgG2c, κ Isotype Ctrl Antibody	Biolegend	Cat# 400721
APC/Cyanine7 Rat IgG2b, κ Isotype Ctrl Antibody	Biolegend	Cat# 400623
Donkey anti-rabbit IgG (H+L) cross-adsorbed secondary antibody, Alexa Fluor 488 (1:1000)	Thermo Fisher Scientific	Cat# A-21206;
Donkey anti-rabbit IgG (H+L) cross-adsorbed secondary antibody, Alexa Fluor 546 (1:1000)	Thermo Fisher Scientific	Cat# A10040;
Donkey anti-rabbit IgG (H+L) cross-adsorbed secondary antibody, Alexa Fluor 647 (1:1000)	Thermo Fisher Scientific	Cat# A-31573;
Donkey anti-goat IgG (H+L) cross-adsorbed secondary antibody, Alexa Fluor 488 (1:1000)	Thermo Fisher Scientific	Cat# A-11055;
Donkey anti-goat IgG (H+L) cross-adsorbed secondary antibody, Alexa Fluor 546 (1:1000)	Thermo Fisher Scientific	Cat# A-11056;
Donkey anti-goat IgG (H+L) cross-adsorbed secondary antibody, Alexa Fluor 647 (1:1000)	Thermo Fisher Scientific	Cat# A-21447;
Donkey anti-mouse IgG (H+L) cross-adsorbed secondary antibody, Alexa Fluor 488 (1:1000)	Thermo Fisher Scientific	Cat# A-21202;
Donkey anti-mouse IgG (H+L) cross-adsorbed secondary antibody, Alexa Fluor 546 (1:1000)	Thermo Fisher Scientific	Cat# A10036;
Donkey anti-mouse IgG (H+L) cross-adsorbed secondary antibody, Alexa Fluor 647 (1:1000)	Thermo Fisher Scientific	Cat# A-31571;
Donkey anti-Guinea Pig IgG (H+L) secondary antibody, Alexa Fluor 647-AffiniPure (1:1000)	Jackson ImmunoResearch Labs	Cat# 706-605-148;
Donkey anti-chicken IgY (IgG) (H+L) secondary antibody, Alexa Fluor 488 (1:1000)	Thermo Fisher Scientific	Cat# A78948;
Cy5-AffiniPure Donkey Anti-Rat IgG (H+L) (1:1000)	Jackson ImmunoResearch Labs	Cat# 712-175-150;
**Drugs, Chemicals, and Kits**		
APCP	Tocris Bioscience	Cat# 3633
NBMPR	Sigma-Aldrich	Cat# N2255
Dipyridamole	Sigma-Aldrich	Cat# D9766
EHNA	Sigma-Aldrich	Cat# E114
Iodotubericidine	Sigma-Aldrich	Cat# I100

Adenosine	Sigma-Aldrich	Cat# A9251
DPCPX	abcam	Cat# ab120396
CPA	abcam	Cat# ab120398
CCPA	Tocris Bioscience	Cat# 1705
NECA	Tocris Bioscience	Cat# 1691
Evans blue	Sigma-Aldrich	Cat# E2129
Lipopolysaccharides from *Escherichia coli* O55:B5	Sigma-Aldrich	Cat# L2880
Sulforhodamine 101	Thermo Fisher Scientific	Cat# S359
Proteome Profiler Mouse Cytokine Array Kit, Panel A	R&D Systems	Cat# ARY006
Proteome Profiler Mouse XL Cytokine Array	R&D Systems	Cat# ARY028
Adenosine Assay Kit (Fluorometric)	abcam	Cat# ab211094
RNeasy Micro Kit	QIAGEN	Cat# 74004
Omniscript RT Kit	QIAGEN	Cat# 205113
Cycloheximide	Sigma-Aldrich	Cat# C7698
DL-Dithiothreitol	Sigma-Aldrich	Cat# D0632
Heparin sodium salt from porcine intestinal mucosa	Sigma-Aldrich	Cat# H5515
Clozapine N-oxide dihydrochloride (CNO)	Tocris Bioscience	Cat# 6329
Tamoxifen	Carbobution	Cat# CC99648
Sucrose	Sigma-Aldrich	Cat# S0389
TRIZMA® base	Sigma-Aldrich	Cat# T1503
Hot Start Taq EvaGreen® qPCR Mix (No ROX)	Axon	Cat# 27490
2-Mercaptoethanol	Sigma-Aldrich	Cat# M3148
Hanks′ Balanced Salt solution	Sigma-Aldrich	Cat# H6648
NP40	Sigma-Aldrich	Cat# 74385
Recombinant RNasin™ Ribonuclease Inhibitor	Promega	Cat# N2515
cOmplete™ Protease Inhibitor Cocktail	Roche	Cat# 11836145001
Dynabeads™ Protein G for Immunoprecipitation	Thermo Fisher Scientific	Cat# 10004D
UltraPure™ 1M Tris-HCl buffer, pH 7.5	Thermo Fisher Scientific	Cat# 15567027
KCl (2 M), RNase free	Thermo Fisher Scientific	Cat# AM9640G
$MgCl_2$ (1 M)	Thermo Fisher Scientific	Cat# AM9530G
PBS, pH7.4	Thermo Fisher Scientific	Cat# 10010023
Tetrodotoxin Citrate (TTX)	Alomone Labs	Cat# T-550
Miglyol® 812	Caesar & Loretz	Cat# 3274
Ketabel 100 mg/ml	bela-pharm	Ketamin
Xylazine 2%	Bayer	Rompun
Buprenorphine	Indivior	Cat# IND00979
Dexamethasone		
TritonX-100	Sigma-Aldrich	Cat# T8787
4′,6-Diamidin-2-phenylindol (DAPI)	Sigma-Aldrich	Cat# D9542
**Mouse lines**		
Mouse: C57BL/6N		N/A
Mouse: $A1AR^{fl/fl}$	(Scammell et al., 2003)	N/A
Mouse: Glast-CreERT2	(Mori et al., 2006)	N/A
Mouse: Cx3cr1-CreERT2	(Jung et al., 2000)	N/A
Mouse: Cspg4-CreERT2	(Huang et al., 2014)	N/A
Mouse: RiboTag mice (Rlp22HA)	(Sanz et al., 2009)	N/A
Mouse: Rosa 26-CAG-lsl-GCAMP3	(Paukert et al., 2014)	N/A
**Viruses**		
AAV2/5 GfaABC1D-$GRAB_{Ado}$ virus	gift from Yulong Li (Peking University, CN)	N/A
rAAV-GFAP-hM4D(Gi)-mCherry-WPREs, AAV2/5	VTA Wuhan	PT-1091
AAV2/5-GFAP-tdtomato virus	gift from André Zeug (Hannover Medical School)	N/A

**Deposited Data**		
Raw and analyzed data	This paper	GSE248275
**Software and Algorithms**		
ImageJ	(Schneideretal., 2012)	https://imagej.nih.gov/ij/
Imaris	Bitplane	https://imaris.oxinst.com/
Zen	Zeiss	https://www.zeiss.com/microscopy/de/produkte/software/zeiss-zen.html
Msparkles	(Stopper et al., 2023)	https://gitlab.com/Gebhard/MSparkles/
GraphPad Prism 10	GraphPad Software	https://www.graphpad.com/
R statistical programming environment	R Foundation for Statistical Computing	https://www.r-project.org/
Bioconductor	(Huber et al., 2015)	https://bioconductor.org/
HISAT2	(Kim et al., 2019)	http://daehwankimlab.github.io/hisat2/
FastQC	(Andrews, 2010)	https://www.bioinformatics.babraham.ac.uk/projects/fastqc/
FeatureCounts	(Liao et al., 2014)	https://rnnh.github.io/bioinfo-notebook/docs/featureCounts.html
DESeq2	(Love et al., 2014)	https://bioconductor.org/packages/release/bioc/html/DESeq2.html
Pheatmap	(Kolde, 2019)	https://cran.r-project.org/web/packages/pheatmap/
ClusterProfiler	(Yu et al., 2012)	https://bioconductor.org/packages/release/bioc/html/clusterProfiler.html
Metascape	(Zhou et al., 2019)	https://metascape.org/
EthoVision XT 11.5	Noldus Technology	https://www.noldus.com/
MATLAB	Mathworks	https://de.mathworks.com/
Igor pro	Wavemetrics	https://www.wavemetrics.com/
Biorender	Biorender	https://www.biorender.com/

Primer sequences

Gene	Primer	Sequence	Purpose
Actb	Forward	CTTCCTCCCTGGAGAAGAGC	RT-qPCR
	Reverse	ATGCCACAGGATTCCATACC	
Cxcl1	Forward	AGACCATGGCTGGGATTCAC	RT-qPCR
	Reverse	CTCGCGACCATTCTTGAGTGT	
Cxcl10	Forward	AAGTGCTGCCGTCATTTTCT	RT-qPCR
	Reverse	GTGGCAATGATCTCAACACG	
Ccl2	Forward	GTTGGCTCAGCCAGATGCA	RT-qPCR

	Reverse	AGCCTACTCATTGGGATCATCTTG	
Ccl5	Forward	TGCCCACGTCAAGGAGTATTT	RT-qPCR
	Reverse	TCTCTGGGTTGGCACACACTT	
Lcn2	Forward	ATGTCACCTCCATCCTGGTC	RT-qPCR
	Reverse	CACACTCACCACCCATTCAG	
Tnf	Forward	CCACCACGCTCTTCTGTCTAC	RT-qPCR
	Reverse	AGGGTCTGGGCCATAGAACT	
Il1a	Forward	CGCTTGAGTCGGCAAAGAAAT	RT-qPCR
	Reverse	CTTCCCGTTGCTTGACGTTG	
Il1b	Forward	TGCCACCTTTTGACAGTGATG	RT-qPCR
	Reverse	TGATGTGCTGCTGCGAGATT	
Il6	Forward	GAGTGGCTAAGGACCAAGACC	RT-qPCR
	Reverse	AACGCACTAGGTTTGCCGA	
Gfap	Forward	TGGAGGAGGAGATCCAGTTC	RT-qPCR
	Reverse	AGCTGCTCCCGGAGTTCT	
Pdgfra	Forward	TCCTTCTACCACCTCAGCGAG	RT-qPCR
	Reverse	CCGGATGGTCACTCTTTAGGAAG	
Pdgfrb	Forward	ATGAATCGCTGCTGGGCGCTCTTC	RT-qPCR
	Reverse	TCAAAGGAGCGGATGGAGTGGTCG	
Itgam	Forward	ATGGACGCTGATGGCAATACC	RT-qPCR
	Reverse	TCCCCATTCACGTCTCCCA	
GCaMP3 KI	Forward	CACGTGATGACAAACCTTGG	genotyping PCR
GCaMP3 KI	Reverse	GGCATTAAAGCAGCGTATCC	
GCaMP3 WT	Forward	CTCTGCTGCCTCCTGGCTTCT	genotyping PCR
GCaMP3 WT	Reverse	CGAGGCGGATCACAAGCAATA	
GLAST	Forward	GAGGCACTTGGCTAGGCTCTGAGGA	genotyping PCR
GLAST KI	Reverse	GGTGTACGGTCAGTAAATTGGACAT	
GLAST WT	Reverse	GAGGAGATCCTGACCGATCAGTTGG	
HA	Forward	GGGAGGCTTGCTGGATATG	genotyping PCR
HA	Reverse	TTTCCAGACACAGGCTAAGTACAC	
A1AR	Forward	CTTTGCCCTCAGCTGGCTACCG	genotyping PCR
A1AR KI	Reverse	ATCGGAATTCGCTAGCTTCGGC	
A1AR WT	Reverse	TTCTCGGGGTCAGGAGAGCACC	
