# Supplementary material for: Adenosine triggers early astrocyte reactivity that provokes microglial responses and drives the pathogenesis of sepsis-associated encephalopathy in mice
Source: Nat Commun. 2024 Jul 27;15:6340. doi: 10.1038/s41467-024-50466-y (PMC11283516; doi:10.1038/s41467-024-50466-y)
Supplement: Supplementary file 2 — Reporting Summary [file 41467_2024_50466_MOESM2_ESM.pdf]

nature portfolio

Corresponding author(s): Wenhui Huang, Frank Kirchhoff

Last updated by author(s): Jun 23, 2024

Reporting Summary

Nature Portfolio wishes to improve the reproducibility of the work that we publish. This form provides structure for consistency and transparency in reporting. For further information on Nature Portfolio policies, see our Editorial Policies and the Editorial Policy Checklist.

Statistics

For all statistical analyses, confirm that the following items are present in the figure legend, table legend, main text, or Methods section.

n/a	Confirmed	
☐	☒	The exact sample size (*n*) for each experimental group/condition, given as a discrete number and unit of measurement
☐	☒	A statement on whether measurements were taken from distinct samples or whether the same sample was measured repeatedly
☐	☒	The statistical test(s) used AND whether they are one- or two-sided<br>*Only common tests should be described solely by name; describe more complex techniques in the Methods section.*
☒	☐	A description of all covariates tested
☐	☒	A description of any assumptions or corrections, such as tests of normality and adjustment for multiple comparisons
☐	☒	A full description of the statistical parameters including central tendency (e.g. means) or other basic estimates (e.g. regression coefficient) AND variation (e.g. standard deviation) or associated estimates of uncertainty (e.g. confidence intervals)
☐	☒	For null hypothesis testing, the test statistic (e.g. *F*, *t*, *r*) with confidence intervals, effect sizes, degrees of freedom and *P* value noted<br>*Give P values as exact values whenever suitable.*
☒	☐	For Bayesian analysis, information on the choice of priors and Markov chain Monte Carlo settings
☒	☐	For hierarchical and complex designs, identification of the appropriate level for tests and full reporting of outcomes
☒	☐	Estimates of effect sizes (e.g. Cohen's *d*, Pearson's *r*), indicating how they were calculated

Our web collection on statistics for biologists contains articles on many of the points above.

Software and code

Policy information about availability of computer code

Data collection	Two-photon laser-scanning microscope (2P-LSM): Custom made setup, ScanImage software;<br>Image acquisition: Zeiss Axioscan, LSM 710, LSM 780, Zeiss Blue Zen, Zeiss Black Zen;<br>Western blot: ChemiDoc-MP (Bio-Rad);<br>Electrophoresis: QUANTUM ST5 Multi-Imaging system (PEQLAB);<br>qRT-PCR: CFX96 (Bio-Rad);<br>Open field test: USB Webcam Camera, NCH Debut Video Capture Software;<br>Electrophysiology: Zeiss Axioskop 2 FS mot, QuantEM 512SC camera (Photometrics), EPC 10 USB amplifier (HEKA), Patchmaster software (HEKA).
Data analysis	2P-LSM Imaging analysis: MSparkles v1.8.19;<br>Image analysis: Zeiss Zen Blue v3.6 for cell counting and cfos intensity quantification;<br>Imaris v9.6: microglia morphology quantification;<br>FIJI: Western blot and electrophoresis;<br>Open field test: EthoVision XT 11.5 (Noldus Technology);<br>Electrophysiology: Matlab;<br>Statistical analysis: Graphpad Prism 9.5.

For manuscripts utilizing custom algorithms or software that are central to the research but not yet described in published literature, software must be made available to editors and reviewers. We strongly encourage code deposition in a community repository (e.g. GitHub). See the Nature Portfolio guidelines for submitting code & software for further information.

Data

Policy information about availability of data

All manuscripts must include a data availability statement. This statement should provide the following information, where applicable:

- Accession codes, unique identifiers, or web links for publicly available datasets
- A description of any restrictions on data availability
- For clinical datasets or third party data, please ensure that the statement adheres to our policy

RNA-seq data generated in this study have been deposited at https://www.ncbi.nlm.nih.gov/geo/query/acc.cgi?acc=GSE248275 accession code GSE248275, and are available with token ofeveywgxlivfwb for peer reviewers. Source data including the list of DESeq2 normalized counts of the RNA-seq are provided with this paper. Further data to support the findings can be obtained upon request to the corresponding authors.

Research involving human participants, their data, or biological material

Policy information about studies with human participants or human data. See also policy information about sex, gender (identity/presentation), and sexual orientation and race, ethnicity and racism.

Reporting on sex and gender	N/A
Reporting on race, ethnicity, or other socially relevant groupings	N/A
Population characteristics	N/A
Recruitment	N/A
Ethics oversight	N/A

Note that full information on the approval of the study protocol must also be provided in the manuscript.

Field-specific reporting

Please select the one below that is the best fit for your research. If you are not sure, read the appropriate sections before making your selection.

☒ Life sciences ☐ Behavioural & social sciences ☐ Ecological, evolutionary & environmental sciences

For a reference copy of the document with all sections, see nature.com/documents/nr-reporting-summary-flat.pdf

Life sciences study design

All studies must disclose on these points even when the disclosure is negative.

Sample size	No statistical methods were used to pre-determine the sample size. All results were collected from 3-20 individual mouse, based on previously published work using the same stimulation paradigm (PMID: 28070126, 33049219, and 34413515). We have utilized all the mice available without bias. The exact sample size is indicated in each figure and figure legend.
Data exclusions	No data were excluded.
Replication	For in vivo experiments, littermates from different breeding pairs were studied and the data were pooled.
Randomization	All data were collected from 3-20 biologically independent littermates (individual mouse), which were assigned to corresponding groups, as noted in the legend of each Figure and Supplementary Figure.
Blinding	The analysis of cell counting, Imaris analysis and behavior were performed in a blind manner. The scientists analyzing the data was not informed about the groups until completion of statistical analyses. For qRT-PCR, RNA-seq and Cytokine array, blinding was not necessary since the values were analyzed by the CT values, reads, and gray intensity, respectively.

Reporting for specific materials, systems and methods

We require information from authors about some types of materials, experimental systems and methods used in many studies. Here, indicate whether each material, system or method listed is relevant to your study. If you are not sure if a list item applies to your research, read the appropriate section before selecting a response.

Materials & experimental systems

n/a	Involved in the study
☐	☒ Antibodies
☒	☐ Eukaryotic cell lines
☒	☐ Palaeontology and archaeology
☐	☒ Animals and other organisms
☒	☐ Clinical data
☒	☐ Dual use research of concern
☒	☐ Plants

Methods

n/a	Involved in the study
☒	☐ ChIP-seq
☒	☐ Flow cytometry
☒	☐ MRI-based neuroimaging

Antibodies

Antibodies used

Goat anti-Sox9 (1:500) R&D Systems Cat# AF3075
Rabbit anti-Iba1 (1:1000) Wako Cat# 019-19741
Goat anti-Iba1 (1:500) abcam Cat# ab5076
Mouse anti-NeuN (1:500) Millipore Cat# MAB377
Rat anti-CD31 (1:100) BD Pharmingen Cat# 550274
Rat anti-Ly6B (1:500) Bio Rad Cat# MCA771GT
Mouse anti-HA (1:500) Biolegend Cat# 901513
Goat anti-LCN2 (1:1000) R&D Systems Cat# AF1857
Chicken anti-GFP (1:1000) Thermo Fisher Scientific Cat# 10524234
Rabbit anti-p-STAT3 (1:1000) Cell Signaling Technology Cat# 9145
Rabbit anti-NF-kappaB p65 (1:500) Cell Signaling Technology Cat# 8242
Guinea pig anti-cFos (1:4000) Synaptic Systems Cat# 226004
Brilliant Violet 421™ anti-mouse CD45 Antibody Biolegend Cat# 103133
APC anti-mouse Ly-6G Antibody Biolegend Cat# 127613
PerCP anti-mouse/human CD11b Antibody Biolegend Cat# 101229
PE/Cyanine7 anti-mouse Ly-6C Antibody Biolegend Cat# 128017
APC/Cyanine7 anti-mouse CD3 Antibody Biolegend Cat# 100221
Brilliant Violet 421™ Rat IgG2b, κ Isotype Ctrl Antibody Biolegend Cat# 400639
APC Rat IgG2a, κ Isotype Ctrl Antibody Biolegend Cat# 400511
PerCP Rat IgG2b, κ Isotype Ctrl Antibody Biolegend Cat# 400629
PE/Cyanine7 Rat IgG2c, κ Isotype Ctrl Antibody Biolegend Cat# 400721
APC/Cyanine7 Rat IgG2b, κ Isotype Ctrl Antibody Biolegend Cat# 400623
Donkey anti-rabbit IgG (H+L) cross-adsorbed secondary antibody, Alexa Fluor 488 (1:1000) Thermo Fisher Scientific Cat# A-21206;
Donkey anti-rabbit IgG (H+L) cross-adsorbed secondary antibody, Alexa Fluor 546 (1:1000) Thermo Fisher Scientific Cat# A10040;
Donkey anti-rabbit IgG (H+L) cross-adsorbed secondary antibody, Alexa Fluor 647 (1:1000) Thermo Fisher Scientific Cat# A-31573;
Donkey anti-goat IgG (H+L) cross-adsorbed secondary antibody, Alexa Fluor 488 (1:1000) Thermo Fisher Scientific Cat# A-11055;
Donkey anti-goat IgG (H+L) cross-adsorbed secondary antibody, Alexa Fluor 546 (1:1000) Thermo Fisher Scientific Cat# A-11056;
Donkey anti-goat IgG (H+L) cross-adsorbed secondary antibody, Alexa Fluor 647 (1:1000) Thermo Fisher Scientific Cat# A-21447;
Donkey anti-mouse IgG (H+L) cross-adsorbed secondary antibody, Alexa Fluor 488 (1:1000) Thermo Fisher Scientific Cat# A-21202;
Donkey anti-mouse IgG (H+L) cross-adsorbed secondary antibody, Alexa Fluor 546 (1:1000) Thermo Fisher Scientific Cat# A10036;
Donkey anti-mouse IgG (H+L) cross-adsorbed secondary antibody, Alexa Fluor 647 (1:1000) Thermo Fisher Scientific Cat# A-31571;
Donkey anti-Guinea Pig IgG (H+L) secondary antibody, Alexa Fluor 647-AffiniPure (1:1000) Jackson ImmunoResearch Labs Cat# 706-605-148;
Donkey anti-chicken IgY (IgG) (H+L) secondary antibody, Alexa Fluor 488 (1:1000) Thermo Fisher Scientific Cat# A78948;
Cy5-AffiniPure Donkey Anti-Rat IgG (H+L) (1:1000) Jackson ImmunoResearch Labs Cat# 712-175-150;

Validation

The primary antibodies have been validated by their manufacturers and literatures, and our own observations agreed with the validation.

Animals and other research organisms

Policy information about studies involving animals; ARRIVE guidelines recommended for reporting animal research, and Sex and Gender in Research

Laboratory animals

This study involved the following animals maintained on C57BL/6 background: C57BL/6 mice; A1AR fl/fl mice(Scammell et al., 2003); Glast-CreERT2(CT2) mice(Mori et al., 2006); Cx3cr1-CreERT2 mice (Jung et al., 2000); NG2-CreERT2 mice (Huang et al., 2014); RiboTag mice (Rlp22HA) (Sanz et al., 2009); GCaMP3 reporter mice (Rosa 26-CAG-lsl-GCAMP3)(Paukert et al., 2014). Both female and male mice were investigated in the study. The age of the mice is 11-13 weeks.

Wild animals

This study did not involve wild animals.

Reporting on sex

Mice of both sexes were used for most experiments (details in supplementary Table S1). We did not observe significant differences

between males and females.

Field-collected samples

The study did not involve samples collected from the field.

Ethics oversight

Animal husbandry and procedures were performed at the animal facility of CIPMM, University of Saarland according to European and German guidelines for the welfare of experimental animals. Animal experiments were approved by the Saarland state's ''Landesamt für Gesundheit und Verbraucherschutz" in Saarbrücken/Germany (animal license number: 65/2013, 12/2014, 34/2016, 36/2016, 03/2021 and 08/2021).

Note that full information on the approval of the study protocol must also be provided in the manuscript.

Plants

Seed stocks

Report on the source of all seed stocks or other plant material used. If applicable, state the seed stock centre and catalogue number. If plant specimens were collected from the field, describe the collection location, date and sampling procedures.

Novel plant genotypes

Describe the methods by which all novel plant genotypes were produced. This includes those generated by transgenic approaches, gene editing, chemical/radiation-based mutagenesis and hybridization. For transgenic lines, describe the transformation method, the number of independent lines analyzed and the generation upon which experiments were performed. For gene-edited lines, describe the editor used, the endogenous sequence targeted for editing, the targeting guide RNA sequence (if applicable) and how the editor was applied.

Authentication

Describe any authentication procedures for each seed stock used or novel genotype generated. Describe any experiments used to assess the effect of a mutation and, where applicable, how potential secondary effects (e.g. second site T-DNA insertions, mosiacism, off-target gene editing) were examined.
