# Supplementary material for: Adenosine triggers early astrocyte reactivity that provokes microglial responses and drives the pathogenesis of sepsis-associated encephalopathy in mice
Source: Nat Commun. 2024 Jul 27;15:6340. doi: 10.1038/s41467-024-50466-y (PMC11283516; doi:10.1038/s41467-024-50466-y)
Supplement: Supplementary file 4 — Description of Additional Supplementary Files [file 41467_2024_50466_MOESM4_ESM.docx]

**Description of Additional Supplementary Files**

**Supplementary Video 1:**

GRABAdo_saline+sr101_application: Injection of saline did not increase the astrocytic GRABAdo1.0 F.I. in vivo. SR101 was supplemented in the injection to indicate the entry of injected solutions into the cerebral blood flow. Scale bar = 50 µm.

**Supplementary Video 2:**

GRABAdo_ado+sr101_application: Increased astrocytic GRABAdo1.0 F.I. was detected when the i.p. injected adenosine reached the blood vessels in the brain indicated by SR101. Scale bar = 50 µm.
